# Salicylaldehyde Benzoylhydrazone Protects Against Ferroptosis in Models of Neurotoxicity and Behavioural Dysfunction, In Vitro and In Vivo

**DOI:** 10.1007/s12031-025-02371-2

**Published:** 2025-06-14

**Authors:** Niamh C. Clarke, Ellen McCabe, Lasse D. Jensen, Bernadette S. Creaven, Derek A. Costello

**Affiliations:** 1 UCD School of Biomolecular & Biomedical Science, University College Dublin, Dublin, Ireland; 2 UCD Conway Institute, University College Dublin, Dublin, Ireland; 3 Department of Medical and Health Sciences, Linköping University, Linköping, Sweden; 4 BioReperia AB, Linköping, Sweden; 5 School of Chemical and BioPharmaceutical Science, Technological University Dublin, Dublin, Ireland

**Keywords:** HT22 cells, Zebrafish larvae, Iron overload, Ferric ammonium citrate, Schiff base, Lipid peroxidation

## Abstract

Metal dyshomeostasis in the brain is a key feature of many neuropathologies, including hypoxic and traumatic injury and chronic conditions such as Alzheimer’s and Parkinson’s disease. Ferroptosis is a form of cell death driven by the intracellular accumulation of iron. This is primarily characterised by a loss in endogenous antioxidant capacity and uncontrolled lipid peroxidation. Ferroptosis has been reported to underlie the pathology associated with several neurological and neurodegenerative conditions and has, therefore, become an attractive target for therapeutic intervention. Salicylaldehyde benzoylhydrazone (SBH) is a specialised hydrazone agent, known for its antibacterial and anticancer properties. It has robust metal-chelating capacity, with a particular affinity for complexing with iron and copper. The current study sought to investigate the potential of SBH to act as an anti-ferroptotic agent and to alleviate the neurotoxic and dysfunctional consequences of iron overload. We demonstrate that SBH can alleviate the death of HT22 hippocampal neurons, induced by exposure to the iron donor, ferric ammonium citrate (FAC). This was accompanied by a reduction in intracellular iron and lipid peroxidation, and alleviation of hallmark changes in gene expression indicative of ferroptosis. Using FAC-incubated zebrafish larvae as an in vivo model of iron overload, we reveal that SBH can reduce the mortality and toxicity associated with FAC exposure. Moreover, we report a FAC-mediated dysfunction in intrinsic sensorimotor reflex behaviour, which is restored by SBH. Taken together, our findings highlight SBH as an anti-ferroptotic agent and support its further investigation as a potential neurotherapeutic for conditions associated with iron dysregulation.

**Supplementary Information:**

The online version contains supplementary material available at 10.1007/s12031-025-02371-2.

## Introduction

Iron is an essential trace element, crucial to the maintenance of cellular homeostasis in the brain. This includes the regulation of integral functions such as oxygen transport, cellular respiration, myelin formation and catecholamine neurotransmitter metabolism. However, iron dysregulation in the brain has been widely reported as a feature within a number of pathological states, including ischaemic injury (Heo et al. 2021; Lee et al. 2025), epilepsy (Chen et al. 2020), neuroinflammatory (McCarthy et al. 2018; Tang et al. 2020) and neurodegenerative disease (Antharam et al. 2012; Ayton et al. 2020; Gao et al. 2025a). Moreover, elevated iron levels in the brain are seen to directly correlate with disease severity (Ayton et al. 2020; Li et al. 2022). Ferroptosis is a recently characterised, iron-dependent form of regulated cell death, distinct from more thoroughly understood mechanisms such as apoptosis, necroptosis and autophagy (Dixon et al. 2012; Li et al. 2020). It has since been widely implicated in the pathology associated with epilepsy (Chen et al. 2020) and neurological diseases such as Alzheimer’s (AD) and Parkinson’s disease (PD) (Lin et al. 2022; Ma et al. 2022; Yao et al. 2024), thus making the study of iron metabolism in the brain and regulation of ferroptosis critical to our understanding of central nervous system (CNS) pathophysiology. Accordingly, the ferroptosis pathway poses an intriguing target for therapeutic intervention into multiple brain pathologies (Chen et al. 2021a; Gao et al. 2025b; Lee et al. 2025).

Ferroptosis is driven by the intracellular accumulation of iron, largely mediated by the transferrin receptor (TfR) (Gao et al. 2015; Herbison et al. 2009). Under pathological conditions, iron can accumulate intracellularly and become reduced to its ferrous (Fe(II)) state. This leads to a loss in the endogenous antioxidant glutathione (GSH) and concomitant inactivation of its effector molecule glutathione peroxidase 4 (GPx4), a key enzyme in the prevention of lipid peroxidation (Yang and Stockwell 2016). This results in the accumulation of lipid hydroperoxides, promotion of reactive oxygen species (ROS) and subsequent oxidative cell death (Chen et al. 2021b; Ursini and Maiorino 2020). Consequently, these features have become hallmark indicators of ferroptosis, coupled with the disruption of molecules critical for the regulation of both fatty acid and iron metabolism, such as acetyl-CoA synthase (ACSL)4 and heme oxygenase-1 (HO-1) (Ursini and Maiorino 2020). Compounds that regulate distinct elements of the ferroptosis pathway have, therefore, shown promise in alleviating neuronal death in vitro and in vivo, using experimental models of neurotoxicity (Cheng et al. 2022; Liu et al. 2024) and CNS conditions such as stroke (Lee et al. 2025; Liao et al. 2025), AD (Lu et al. 2025; Wu et al. 2025) and multiple sclerosis (Seke et al. 2025).

Schiff base compounds are molecules produced by condensation reactions between amine and carbonyl groups, forming auxiliary ligands with affinity for transition metal ions, revealing important bioactivity, such as antiproliferative and cytotoxic effects (Adeleke et al. 2021; Deng et al. 2019; Hu et al. 2018; Md Yusof et al. 2015). Hydrazones are a subset of Schiff bases, containing an additional hydrazine group. Hydrazones and their derivatives are known to possess an array of biological activities and are routinely used for agricultural purposes due to their antimicrobial effects (Raczuk et al. 2022). However, their medicinal properties, such as their application as antibacterial and anticancer therapeutics, are becoming increasingly recognised (Altamimi et al. 2024; El Rayes et al. 2022). These include properties pertinent to the CNS, such as anti-convulsant, anti-inflammatory and antioxidant effects (Rollas and Kucukguzel 2007; Tok et al. 2021). Hydrazones have been widely used in analytical chemistry as complexation reagents for transition metal ions such as iron, zinc, copper and cobalt, and their capacity to form strong metal complexes is considered central to their wide range of biological effects (Narang et al. 2012; Verma et al. 2014). Their selectivity towards complexing with a specific cation is largely dependent on physiological ion concentration within a given biological system. As the metabolism of copper(II) and iron(II) is intrinsically linked in mammalian cells, the chelation of one will, therefore, have a distinct impact on the regulation of the other, thus contributing to the hydrazone sphere of influence (Aigner et al. 2008; Doguer et al. 2018). This is particularly pertinent to the brain, in which an imbalance of both copper and iron is known to contribute to the protein misfolding and ROS generation that underlie multiple neurodegenerative disease pathologies (Chen et al. 2025).

Salicylaldehyde benzoylhydrazone (SBH) is a specialised hydrazone with robust chelating capacity, coupled with a high degree of structural stability and solubility. Derivatives of SBH have previously revealed potential as anticancer agents, based on their potent anti-proliferative effects (Hristova-Avakumova et al. 2017; Nikolova-Mladenova et al. 2025). The anti-radical and anti-inflammatory effects of SBH and its structural analogues have also recently been investigated by several groups, with the anti-radical activity ascribed to its ability to act both as an antioxidant and as an iron chelator (Boyazhieva et al. 2019; Hristova-Avakumova et al. 2021). However, despite this, the potential modulatory effects of SBH in brain disorders have received limited exploration to date. The primary aim of the current study was to evaluate the neuroprotective capacity of SBH. In particular, we sought to examine the impact of SBH on iron-induced neuronal cell death and regulation of ferroptosis in vitro*,* using hippocampal HT22 cells exposed to ferric ammonium citrate (FAC) (Cheng et al. 2022; Liu et al. 2024; Park et al. 2015). Larvae from zebrafish (*Danio rerio*) stimulated with FAC have previously proven to be a reliable in vivo, vertebrate model of iron overload (Nasrallah et al. 2018). Using this model, we further explored the ability of SBH to alleviate iron-mediated toxicity and restore behavioural integrity in vivo.

## Methods

### Maintenance and Treatment of HT22 Cells

Murine HT22 hippocampal neurons (CLS, Germany) were maintained in Dulbecco’s modified Eagle’s medium (DMEM; Gibco), supplemented with 10% (v/v) foetal bovine serum (FBS) and 1% penicillin:streptomycin (Gibco, UK) at 37 °C and 5% CO_2_. Cells were either plated in 96-well plates at a density of 2500 cells/well or plated in 12-well plates at a density of 200,000 cells/well.

Salicylaldehyde benzoyl hydrazone (SBH; Suppl.1) was solubilised in dimethylsulfoxide (DMSO; Sigma-Aldrich, UK) to a 40 mM stock solution. Ferric ammonium citrate (FAC; Honeywell, USA) was solubilised in sterile dH_2_O to a 40 mM stock. Concentration–response analyses were carried out on HT22 cells in 96-well plates using increasing concentrations of SBH (0–100 µM) or FAC (0–600 µM). HT22 cells were plated in either 96-well plates or 12-well plates and incubated with FAC (75 µM) in the presence and absence of SBH (10 µM) for 24 h. The equivalent volume of DMSO was used as a vehicle control for SBH. Cells were assessed for viability and cytotoxicity or harvested for subsequent RNA extraction or protein quantification.

### Cell Viability and Cytotoxicity Analysis

HT22 cell viability was assessed using the Cell Counting Kit-8 (CCK-8; Dojindo Labs, USA) as per the manufacturer’s instructions. Briefly, the supernatant was removed and replaced with CCK-8 reagent, diluted 1:10 in DMEM (100 µl/well). Cells were incubated at 37 °C for 1 h. The supernatant was harvested (50 µl/well) and removed to the corresponding wells of a new 96-well plate. Absorbance was read at 450 nm. Cytotoxicity was evaluated using the lactate dehydrogenase (LDH) assay (MedChemExpress, USA) according to the manufacturer’s guidelines. Following treatment, the cell supernatant was removed to the corresponding wells of a new 96-well plate (50 µl/well) and incubated with an equal volume of LDH reaction buffer (30 min, room temperature). The assay stop solution was applied to stop the reaction, and absorbance was read at 490 nm. Optical density was determined using a SpectraMax M3 plate reader and SoftMax Pro 6.2.1 software (Molecular Devices, USA). Data is expressed as a percentage of the control values for each experiment.

### Iron Concentration and Lipid Peroxidation Quantification Analysis

HT22 cells were harvested using radioimmunoprecipitation assay (RIPA) buffer (pH 8; containing: NaCl 150 mM, 0.5% sodium deoxycholate, Tris 50 mM, 0.1% sodium dodecyl sulphate, 1% IGEPAL) supplemented with 1:100 protease inhibitor cocktail (Merck, UK). Samples were normalised to 1 µg/µl protein, following a bicinchoninic acid assay (BCA) assay (Thermo Fisher Scientific, UK). The total iron concentration in cell lysates was determined using a colorimetric Iron Assay kit (ScienCell, USA), in accordance with the manufacturer’s guidelines. Briefly, iron standards (0–400 µM) and cell lysates (10 µg protein) were diluted in assay buffer and added to duplicate wells of a 96-well plate. Working reagent was applied to each well (200 µl) and incubated in the dark for 30 min. Absorbance was read at 590 nm, and total iron concentration was calculated according to a calibration curve. Peroxides were measured using PEROXsayTM‐LIPID kit (G-Biosciences, USA). Hydrogen peroxide standards (0–50 µM) and cell lysates (10 µg protein) were applied to duplicate wells of a 96-well plate and incubated in assay solution (100 µl) for 30 min. Absorbance was read at 595 nm, and peroxide content was calculated from the standard curve. Both iron and lipid peroxidation are expressed as µM/µg protein.

### Gene Expression Analysis

HT22 cells were harvested using E.Z.N.A.® Total RNA Kit I (Omega Bio-tek, UK), according to the manufacturer’s instructions. RNA was eluted in 20 µl of nuclease-free water (Invitrogen, UK), quantified using a NanoDrop™ 2000 Spectrophotometer (Thermo Scientific, UK), and stored at − 80 °C. cDNA was synthesised from 500 ng of RNA per sample, using High-Capacity cDNA Reverse Transcription Kit (Thermo Fisher, UK). cDNA was stored at − 20 °C. Expression of Gpx4, Hmox1, Acsl4 and Actb mRNA was analysed by quantitative real-time PCR on a QuantStudio 7Flex qPCR System. SYBR™ Green PCR Master Mix (Applied Biosciences, UK) was used in each reaction, along with primers for equivalent mouse genes (Table 1; KiCqStart; Sigma-Aldrich, UK). Quantification cycle (Ct) values were determined for individual samples, and the relative gene expression was expressed using the comparative Ct (ΔΔCt) method.

**Table 1:** Primer sequences

Species	Gene	Forward primer (5′ → 3′)	Reverse primer (5′ → 3′)
Mouse	*Acsl4*	GTTCCGGAAATCATGGATAG	CAGTATCAGATTACAAAGAGGG
Mouse	*Actb*	GATGTATGAAGGCTTTGGTC	TGTGCACTTTTATTGGTCTC
Mouse	*Gpx4*	TGGATAAGTACAGGGGTTTC	TAGCTGAGTGTAGTTTACGTC
Mouse	*Hmox1*	CATGAAGAACTTTCAGAAGGG	TAGATATGGTACAAGGAAGCC

### Zebrafish Maintenance and Treatment

In vivo experiments were carried out on zebrafish (*Danio rerio*) larvae up to a maximum of 5 days post-fertilisation (dpf) in accordance with European Directive 2010/63/EU. Embryos were generated through the natural spawning of adult Tg(fli1a:egfp) transgenic zebrafish to express EGFP under the control of the Fil1 promotor (Lawson and Weinstein 2002). Embryos were maintained at 28.5 °C in 1X embryo medium (containing (in mM): 4.96 NaCl, 0.18 KCl, 0.33 CaCl_2_*2H_2_O and 0.4 MgCl_2_*6H_2_O; pH 7.2) supplemented with 1X (0.003%) 1-phenyl-2-thiourea (PTU, Sigma-Aldrich) to limit pigmentation.

To determine the effect of FAC on zebrafish larvae in vivo, 4dpf larvae were incubated with FAC (0–600 µM) and applied to the embryo media for 24 h. To examine the further impact of SBH, 4dpf zebrafish larvae were anesthetised with tricaine (5 mg/ml) and transferred to an agarose plate (2% w/v). SBH (10 µM) or vehicle control (0.025% DMSO), prepared in embryo medium supplemented with PTU, was injected into the caudal vein using the microINJECTOR™ All-Digital Multi-pressure System (Tritech Research). Fish were allowed to recover for 30 min in embryo medium before being randomly assigned to either normal embryo medium or embryo medium containing FAC for 24 h at 28.5 °C (15 larvae per group with the experiment performed in triplicate) in an individual well of a 24-well plate.

### Analysis of Zebrafish Viability and Activity

Viability was assessed as the percentage of surviving larvae per group, 24 h following treatment. Dead larvae were determined by a lack of detectable heartbeat, coupled with a loss of tissue transparency and/or visible signs of tissue necrosis. The presence of non-lethal malformations was recorded per larva and classified in accordance with the criteria adapted from Bar-Ilan and colleagues (2009) (Table 2). Individual larvae were scored between 0 and 4, indicative of the number of morphological malformations. The touch startle response was assessed as a measure of inherent reflex behaviour in surviving larvae. Briefly, larvae were startled by light mechanical stimulation to the tail with a dissection teasing probe. A positive startle response was recorded as an escape-like darting movement away from the stimulus. The proportion of positive startle responses was recorded per group.

**Table 2:** Classification of non-lethal malformations in zebrafish larvae (5dpf)

Class	Classification criteria
0	A fish with normal morphology
1	Fish with a minor change to their morphology
2	Fish with 2 changes in their morphology
3	Fish with multiple ≥ 3 major changes to their morphology
4	Dead fish

### Statistical Analysis

Statistical comparisons across multiple groups were made using one-way analysis of variance (ANOVA). Two-way ANOVA was used to assess the effects and interactions between the two independent variables. Bonferroni post hoc analysis was carried out to identify changes between specific groups. Chi-squared analysis was used to test proportional changes in groups of zebrafish larvae. All graphs and statistical analysis were carried out using GraphPad Prism 10 software (**p < 0.01, ***p < 0.001 and ****p < 0.0001).

## Results

### SBH Protects Against FAC-Induced Neuronal Cell Death

**Fig. 1:**
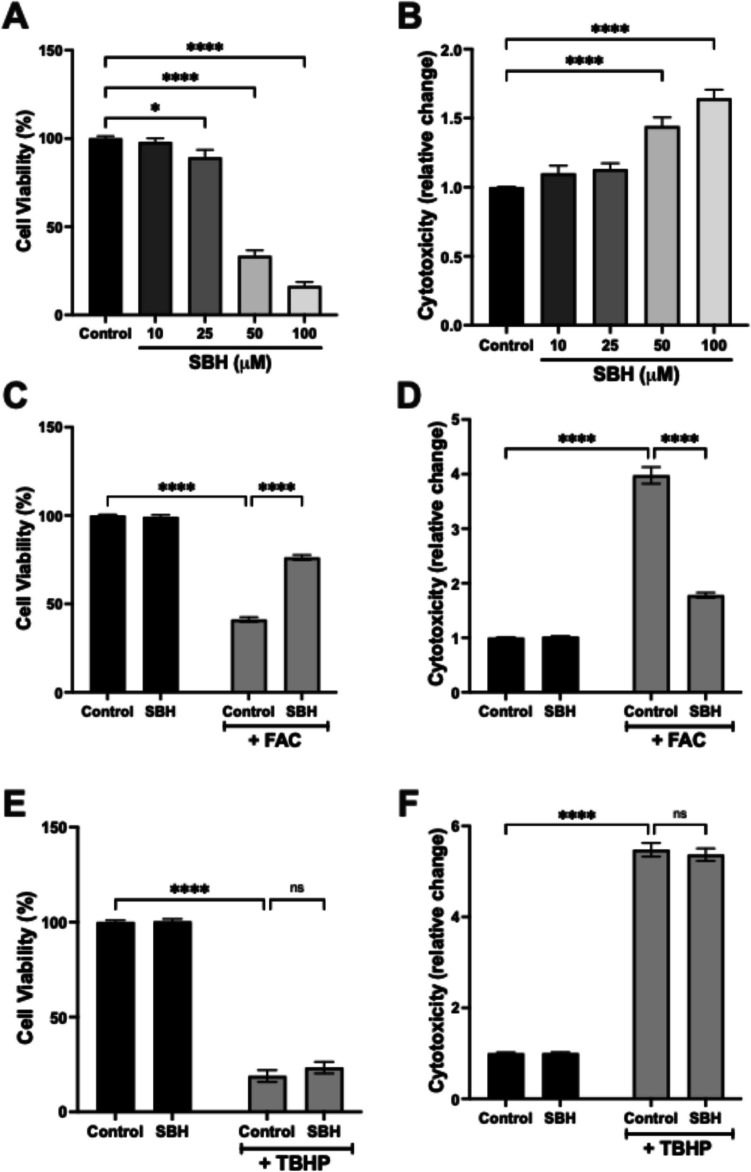
SBH significantly alleviates FAC-induced loss of neuronal cell viability and cytotoxicity. Cell viability (**A**, **C**, **E**) and cytotoxicity (**B**, **D**, **F**) were evaluated in HT22 hippocampal neuronal cells exposed to either SBH alone (0–100 µM; **A**, **B**), FAC (75 µM; **C**, **D**) or TBHP (200 µM; **E**, **F**) for 24 h, in the presence of SBH (10 µM) or DMSO as a vehicle control. Data is presented as mean ± SEM. *p < 0.05, ****p < 0.0001, one- or two-way ANOVA followed by Bonferroni post hoc analysis. *n* = 12 replicates from 4 independent experiments

To assess the neuronal tolerance to SBH, hippocampal HT22 cells were incubated with increasing concentrations of SBH (0–100 µM) for 24 h. A significant loss of cell viability was recorded following exposure to SBH at 25, 50 and 100 µM (*p* < 0.05, *p* < 0.001, *p* < 0.0001, respectively), which was not observed in response to 10 µM (Fig. 1A). At both 50 and 100 µM, this was accompanied by the release of LDH, indicative of cytotoxicity (*p* < 0.0001, one-way ANOVA; Fig. 1B). Accordingly, 10 µM was determined as the optimal working concentration for SBH, revealing no evidence of neuronal toxicity. A preliminary investigation of the concentration-dependent relationship between hippocampal HT22 cell viability and FAC exposure revealed an IC_50_ value of 67.7 µM (Suppl. 2). To investigate the impact of SBH on FAC-induced neuronal death, HT22 cells were incubated with FAC (75 µM) for 24 h, in the presence and absence of SBH (10 µM). Exposure to FAC led to a significant reduction in cell viability (*p* < 0.0001; Fig. 1C) and a concomitant increase in cytotoxicity (*p* < 0.0001, two-way ANOVA; Fig. 1D). In each case, co-incubation with SBH significantly restored the FAC-induced changes (*p* < 0.0001; two-way ANOVA; Fig. 1A, B, respectively).

A similar reduction in HT22 cell viability (*p* < 0.0001; Fig. 1E) and an increase in cytotoxicity (*p* < 0.0001; Fig. 1F) were identified in response to the application of the oxidative stressor tert-butyl hydroperoxide (TBHP; 200 µM; 24 h). Interestingly, however, incubation with SBH did not significantly alter these measures of cell death, compared with cells exposed to TBHP alone (two-way ANOVA; Fig. 1E, F). These findings suggest that SBH can selectively mitigate FAC-induced neuronal death over oxidative stress-mediated apoptosis.

### SBH Reduces Ferroptosis in HT22 Hippocampal Neurons

**Fig. 2:**
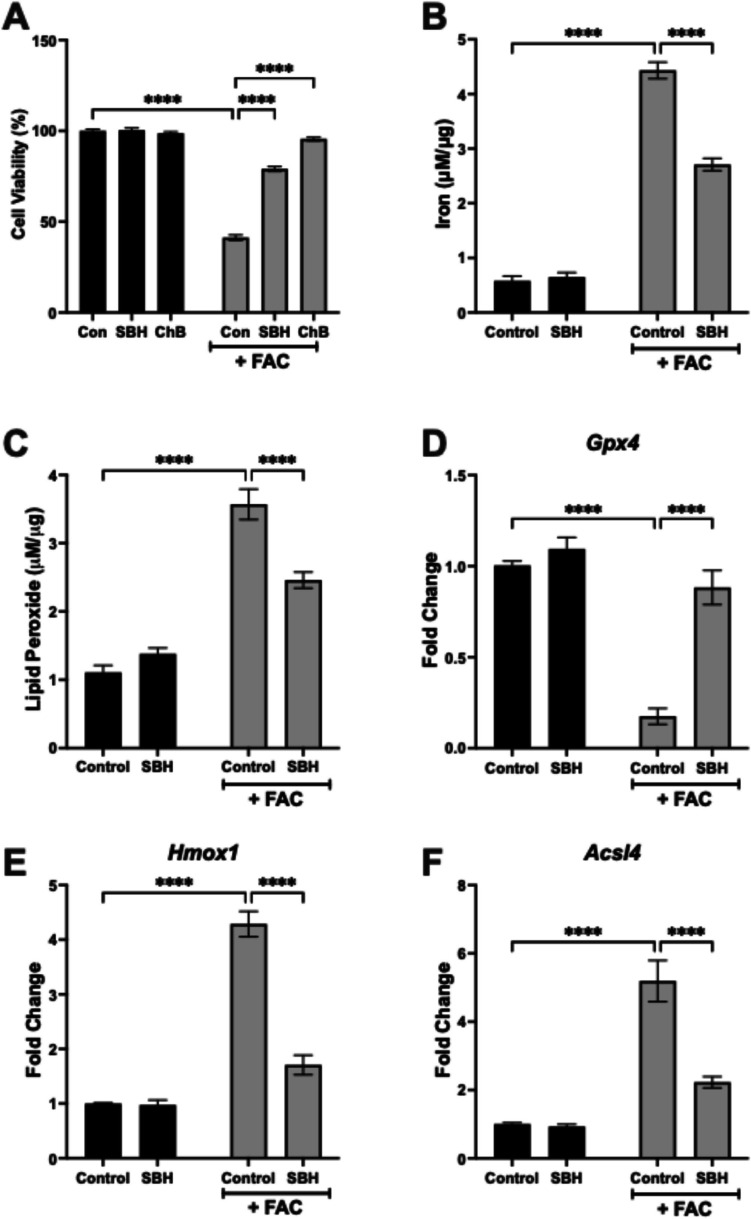
SBH attenuates markers of ferroptosis. HT22 hippocampal neurons were exposed to FAC (75 µM) for 24 h, in the presence and absence of SBH (10 µM) or ChB (50 µM). Cell viability was assessed by CCK-8 assay (**A**). Iron accumulation (**B**) and lipid peroxidation (**C**) were determined in HT22 cell protein lysates. The mRNA expression of *Gpx4* (**D**), *Hmox1* (**E**), and *Acsl4* (**F**) was determined as key indicators of ferroptosis. Data is presented as mean ± SEM. ****p < 0.0001, two-way ANOVA and Bonferroni analysis. *n* = 9 replicates from 3 independent experiments

The agent ferristatin-II/chlorazol black (ChB) has previously been reported as a potent inhibitor of ferroptosis in HT22 cells, through inhibition of TfR-mediated iron uptake (Cheng et al. 2022). To validate the role of ferroptosis in FAC-mediated cell death, HT22 cells were incubated with FAC in the presence of either SBH (10 µM) or ChB (50 µM). These findings revealed that the FAC-induced loss in HT22 cell viability was prevented in the presence of ChB (*p* < 0.0001; Fig. 2A), supporting a role for TfR in mediating neuronal death. Accordingly, 24 h exposure to FAC was coupled with a significant increase in intracellular iron content of HT22 cells (*p* < 0.0001; Fig. 2B), which was further associated with an increase in lipid peroxidation (*p* < 0.0001; Fig. 2C). The presence of SBH significantly attenuated both the FAC-induced increase in iron (*p* < 0.0001) and lipid peroxidation (*p* < 0.0001) when compared to cells exposed to FAC alone (Fig. 2B, C, respectively). Despite reducing FAC-induced iron influx, however, an excess of intracellular iron was still measured in cells stimulated with FAC + SBH, relative to controls (4.7 ± 0.2-fold increase from control; *p* < 0.0001; Fig. 2B).

To further confirm SBH as an anti-ferroptosis agent, we assessed FAC-treated HT22 cells for the expression of key genes involved in the ferroptotic pathway. Consistent with their role in ferroptosis, incubation with FAC significantly reduced the expression of *Gpx4* (*p* < 0.0001; Fig. 2D), alongside an increase in both *Hmox1* (*p* < 0.0001; Fig. 2E) and *Acsl4* (*p* < 0.0001; Fig. 2F). In each case, the FAC-induced change was significantly alleviated in cells co-incubated with SBH, compared with those treated with FAC alone (*p* < 0.0001, two-way ANOVA; Fig. D, E, F, respectively). Taken together, these findings highlight that SBH can protect against ferroptosis-mediated neuronal death.

### Ferroptosis-Mediated Toxicity In Vivo and Behavioural Dysfunction Is Alleviated by SBH

**Fig. 3:**
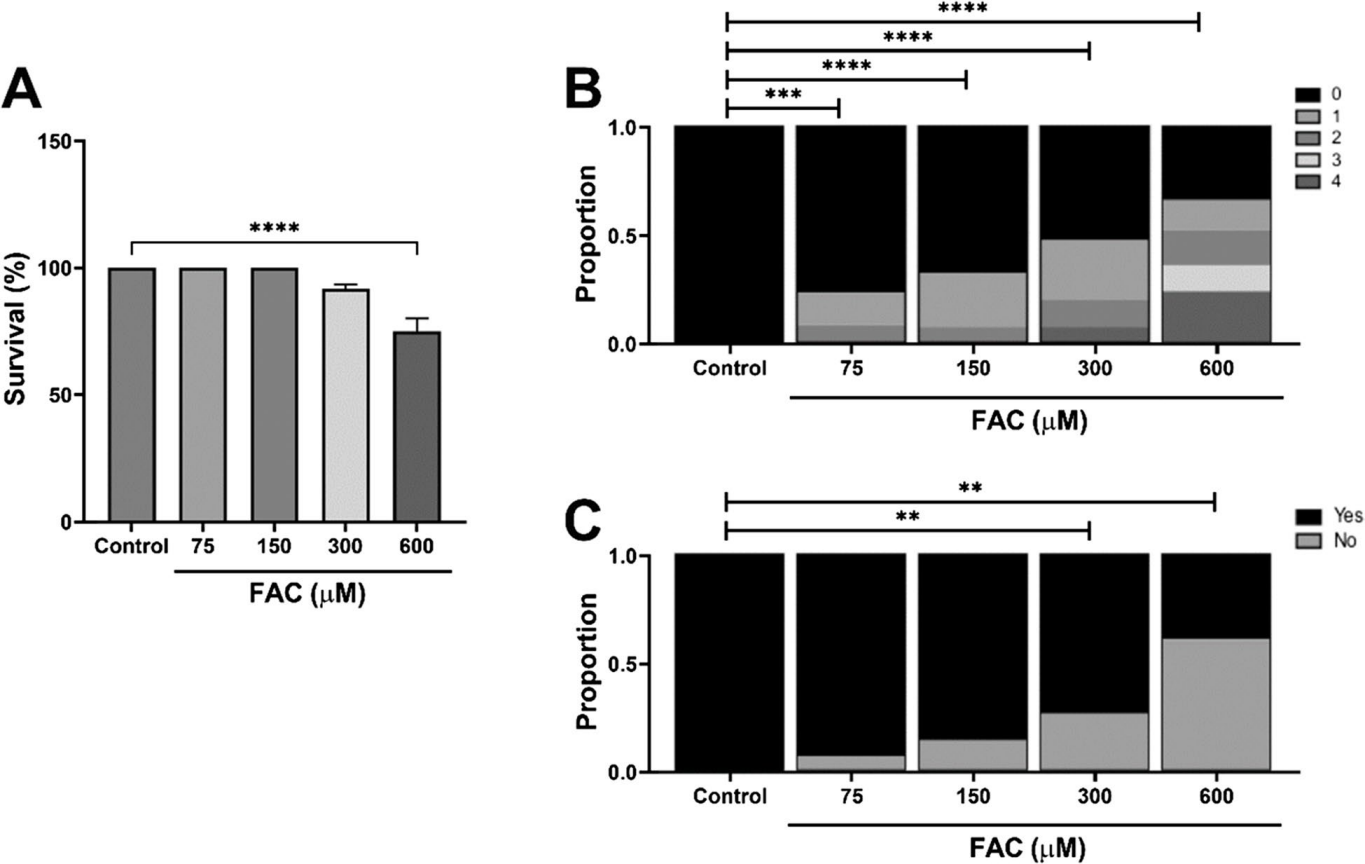
FAC induces a loss of morphological and behavioural integrity in zebrafish larvae in vivo. Zebrafish larvae (4 dpf) to FAC (0–600 µM) for 24 h. Individual larvae were assessed for survival (**A**), incidence of non-lethal malformations (**B**) and touch startle response (**C**). Data presented as mean ± SEM (**A**) or as a proportion of total larvae (**B**, **C**). Statistical comparisons were made by one-way ANOVA or Chi-squared analysis. **p < 0.01, ***p < 0.001, ****p < 0.0001. *n* = 45 larvae from 3 independent experiments

Having revealed the restorative effect of SBH on ferroptosis-mediated neuronal integrity, we sought to develop an in vivo model of iron-mediated dysfunction. Zebrafish larvae (4 dpf) were incubated with increasing concentrations of FAC (75, 150, 300 and 600 µM), in a similar manner to previously reported protocols (Nasrallah et al. 2018). Larval survival and morphological malformations were assessed 24 h following exposure as indicators of toxicity. Survival was significantly reduced in response to FAC 600 µM, compared to untreated controls (*p* < 0.0001, one-way ANOVA; Fig. 3A); an effect which was not observed following exposure to lower concentrations. However, FAC increased the proportion of larvae presenting with non-lethal morphological malformations (Table 2) in a concentration-dependent manner when compared with controls (*p* < 0.001, *p* < 0.0001, Chi-squared analysis; Fig. 3B). Moreover, larvae exposed to FAC at either 300 or 600 µM experienced a significant loss in the touch startle reflex response, indicative of FAC-induced sensory and locomotor dysfunction (*p* < 0.01, Chi-squared analysis; Fig. 3C).

**Fig. 4:**
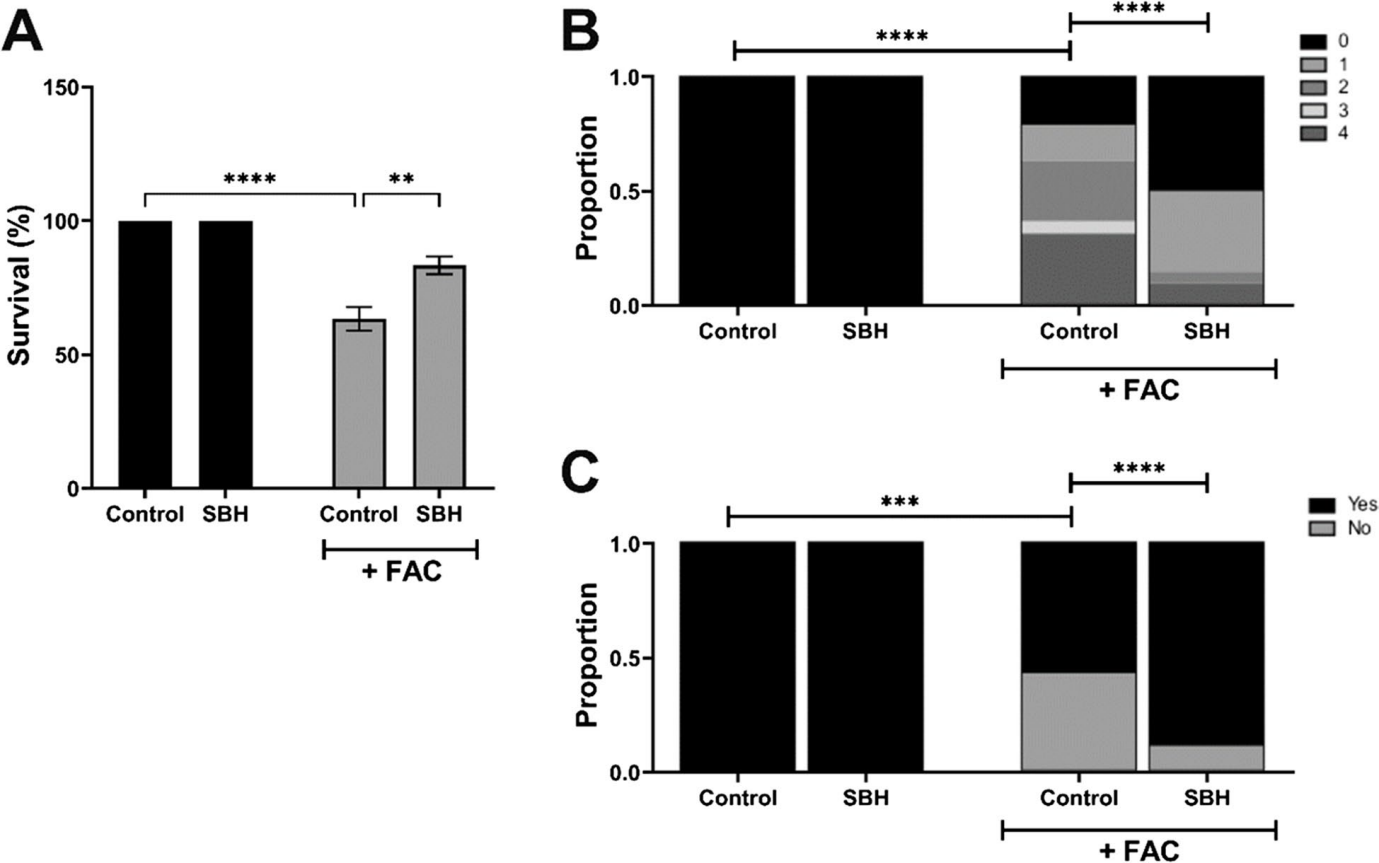
SBH significantly protects against FAC-induced toxicity in zebrafish *larvae* in vivo. Zebrafish larvae (4 dpf) were injected i.v. with SBH (10 µM) or vehicle, in the presence and absence of bath-applied FAC (600 µM; 24 h). Larvae were assessed for survival (**A**), incidence of non-lethal malformations (**B**) and touch startle response (**C**). Data presented as mean ± SEM (**A**) or as a proportion of total larvae (**B**, **C**). Statistical comparisons were made by two-way ANOVA or Chi-squared analysis. **p < 0.01, ***p < 0.001, ****p < 0.0001. *n* = 45 larvae from 3 independent experiments

To examine the impact of SBH on ferroptosis-induced toxicity in vivo, SBH (10 µM) or vehicle was administered to larvae (4 dpf) via i.v. injection to the caudal vein. To evoke iron-mediated dysfunction, FAC (600 µM) was subsequently applied to the bathing media for 24 h. Injection of SBH alone revealed no adverse effects on either larval survival (Fig. 4A) or non-lethal malformations (Fig. 4B), compared with untreated controls. However, treatment with SBH significantly improved survival of FAC-treated larvae, compared with fish exposed to FAC alone (*p* < 0.01, two-way ANOVA; Fig. 4A). In addition, the incidence of non-lethal malformations was reduced in SBH-treated larvae (*p* < 0.0001, Chi-squared analysis; Fig. 4B), which was accompanied by a restoration of the touch startle response (*p* < 0.0001, Chi-squared analysis; Fig. 4C). Collectively, these findings reveal that SBH can effectively mitigate against the in vivo toxicity and behavioural dysfunction characteristic of ferroptosis.

## Discussion

The bioactive effects of hydrazones have been widely reported, particularly with regard to their anticancer and anti-proliferative capacity (Altamimi et al. 2024; El Rayes et al. 2022). Structural analogues of SBH have also revealed anti-proliferative abilities, coupled with their radical scavenging functions (Hristova-Avakumova et al. 2017; Nikolova-Mladenova et al. 2025). Existing evidence suggests that the bioactivity of SBH centres primarily on (i) its capacity for metal chelation and (ii) its lipophilic antioxidant effects. Moreover, the radical scavenging capacity of SBH is thought to be independent of its iron-chelating ability (Hristova-Avakumova et al. 2018). Here, we report that SBH can reduce the viability of hippocampal HT22 neurons in a concentration-dependent manner. This is accompanied by a concomitant increase in LDH release, confirming this to be SBH-mediated cytotoxicity rather than reduced cell proliferation. However, at a non-toxic concentration, we also reveal that SBH is protective against neuronal death mediated by iron overload. In contrast to this, a protective effect was not seen in HT22 cells following exposure to the organic peroxide TBHP. This highlights a selectivity for iron-induced cell death over peroxide-mediated apoptosis, which is surprising considering the widely reported antioxidant abilities of SBH and its analogues (Hristova-Avakumova et al. 2021). Excess iron participates in the Fenton reaction, which promotes the generation of ROS, in particular hydroxyl radicals, and subsequent initiation of lipid peroxidation (Chen et al. 2025; Yang and Stockwell 2016). Indeed, the small-molecule ferroptosis inhibitor, ferrostatin-1, is known to target lipid ROS but has also proven to be ineffective at rescuing apoptosis-induced cell death (Dixon et al. 2012). It is likely, therefore, that SBH can mitigate iron(II)-mediated generation of lipid-derived ROS but cannot protect against caspase-dependent apoptosis. This is particularly interesting as previous observations have demonstrated that derivatives of SBH conveyed protection against NMDA receptor-mediated excitotoxicity in differentiated neuroblastoma cells (Hristova-Avakumova et al. 2017); a process that is largely governed by superoxide production, but can occur in a caspase-independent manner (Minnella et al. 2018; Wang et al. 2004). Moreover, many classical anti-apoptotic agents have previously been found to be ineffective against ferroptosis-mediated cell death (Dixon et al. 2012). We can speculate, therefore, that SBH may be effective at alleviating ferroptotic, excitotoxic and other caspase-independent mechanisms of cell death (Cande et al. 2002; Oliveira et al. 2021), consistent with the complexity of neurodegenerative disease states.

Our findings also support existing evidence that the FAC-induced neuronal death is driven by iron overload since inhibition of TfR prevented the cell loss (Byrne et al. 2013). Accordingly, we found that SBH mitigated cellular iron content in the presence of FAC, along with the consequent increase in lipid peroxidation that typifies ferroptosis. This was further accompanied by an SBH-mediated restoration in the expression of *Gpx4* in conjunction with key regulatory genes along the ferroptotic pathway. The current study did not directly evaluate the iron-chelating capacity of SBH in HT22 cells. We, therefore, cannot rule out its potential impact on other metal ions, such as copper or zinc, which are highly regulatory in neuronal cells (Chen et al. 2025). However, since total iron accumulation was alleviated in FAC-stimulated cells, in addition to downstream changes associated with iron overload, it is reasonable to assume that SBH directly influences this initial driver of ferroptosis, rather than selectively targeting its downstream effectors (Yang and Stockwell 2016). Taken together, these findings offer compelling evidence to support the neuroprotective capacity of SBH as a neuroprotective agent, as a consequence of its anti-ferroptotic activity.

In recent years, zebrafish have become an attractive experimental tool for modelling human disease. In particular, they have proven effective at recapitulating specific features of brain pathologies, including epilepsy (Paudel et al. 2020; Shaw et al. 2022) and neurodegenerative disorders (Bashirzade et al. 2022; Luo et al. 2024; Nada et al. 2016), along with developmental and age-associated changes (Liu 2023). The oxidative stress and toxicity associated with iron dyshomeostasis have been documented in zebrafish (Camiolo et al. 2019), and FAC-treated larvae have been recognised as a reliable model for evaluating secondary iron overload in vivo (Nasrallah et al. 2018). Indeed, a recent study has reported the expression of key ferroptosis hallmarks in FAC-treated larvae, including lipid peroxidation and impaired GPX4 activity (Yan et al. 2025). Rather than repeating this analysis, we focused on characterising the morphological and behavioural phenotype associated with the iron-mediated toxicity in FAC-exposed zebrafish larvae. We highlight the dose-dependent induction of morphological malformations in larvae, which culminates in an increase in the rate of mortality. This is also accompanied by a reduction in the touch startle reflex, indicative of a loss of inherent sensory or neuromuscular integrity. Importantly, however, these changes were alleviated by treatment with SBH, which led to a reduction in mortality and the proportion of morphological anomalies presenting in FAC-stimulated larvae and a significant restoration of the startle reflex. Our current findings, therefore, support our in vitro analyses, highlighting the capacity of SBH to mitigate the toxicity and dysfunction resulting from iron-mediated ferroptosis.

A substantial body of evidence has emerged in recent years, describing the diverse roles of metal ion dyshomeostasis in neurodegenerative pathology. This extends to the pathology associated with environmental stress, in which heavy metals such as lead, copper, cadmium and nickel can promote toxicity (Madesh et al. 2024; Nguyen et al. 2020; Sarkar et al. 2025; Wang et al. 2023). Zebrafish have proven highly useful in these investigations, and indeed recent studies have revealed the integral role of ferroptosis in mediating the developmental and neurotoxic consequences of metal exposure (Cao et al. 2025; Wang et al. 2023). Accordingly, the repurposing of metal chelators and the development of novel metal-targeting agents have gained considerable interest for their potential as neurotherapeutics (Savelieff et al. 2019). We recognise that SBH may restrict iron influx in vitro through direct chelation of FAC extracellularly and thus reduce its bioavailability. In vivo, however, SBH was administered intravenously to zebrafish larvae, which eliminates the possibility of direct chelation with bath-applied FAC. This further supports the suggestion that SBH targets iron accumulation within tissues and cytosol, rendering it non-reactive and thus alleviating ferroptosis. It is also likely that SBH further promotes neuroprotection through membrane radical scavenging, although we have not directly interrogated this here. Taken together, the findings of the current study offer compelling evidence for the use of SBH as an anti-ferroptotic agent and support further investigation of its bioactivity in the context of neurodegeneration. Coupled with existing evidence of the anti-inflammatory properties of hydrazones (Rollas and Kucukguzel 2007), SBH and its derivatives may offer potential as therapeutics for neurodegenerative disease and complex neurotoxic conditions.

## Supplementary Information

Below is the link to the electronic supplementary material.

Supplementary file1 (DOCX 84 KB)

## Data Availability

Data is contained within the article and supplementary information.
