# Supplementary material for: Salicylaldehyde Benzoylhydrazone Protects Against Ferroptosis in Models of Neurotoxicity and Behavioural Dysfunction, In Vitro and In Vivo
Source: J Mol Neurosci. 2025 Jun 14;75(2):77. doi: 10.1007/s12031-025-02371-2 (PMC12167254; doi:10.1007/s12031-025-02371-2)
Supplement: Supplementary file 1 — Supplementary file1 (DOCX 84 KB) [file 12031_2025_2371_MOESM1_ESM.docx]

**SUPPLEMENTARY MATERIAL**

**Supplementary Figure 1**

**Suppl. 1: Structure of salicylaldehyde benzoylhydrazone (SBH).** Image created using ChemDraw software (Perkin Elmer Informatics, USA).

**Supplementary Figure 2**


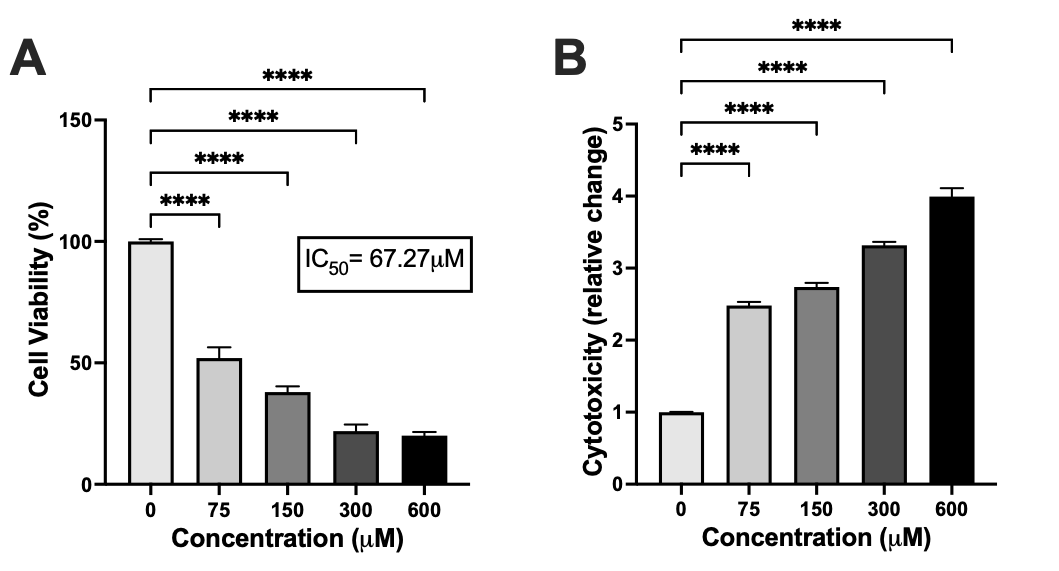


**Suppl. 2: FAC promotes loss of neuronal cell viability and enhances cytotoxicity in a concentration-dependent manner.** Cell viability (A) and cytotoxicity (B) were evaluated in HT22 hippocampal neuronal cells exposed to FAC (0-600µM) for 24h. IC_50_ value (inset) represents the concentration of FAC required to induce a 50% loss of cell viability. Data is expressed as a proportion of control cells, and is presented as mean ± SEM. ****p<0.0001, one-way ANOVA followed by Bonferroni post hoc analysis. n=12 replicates from 4 independent experiments.
